# Dissecting the Re-Os molybdenite geochronometer

**DOI:** 10.1038/s41598-017-16380-8

**Published:** 2017-11-22

**Authors:** Fernando Barra, Artur Deditius, Martin Reich, Matt R. Kilburn, Paul Guagliardo, Malcolm P. Roberts

**Affiliations:** 1 Department of Geology and Andean Geothermal Center of Excellence (CEGA), FCFM, Universidad de Chile, Plaza Ercilla 803, Santiago, Chile; 2 School of Engineering and Information Technology, Murdoch University, 90 South Street, Murdoch, Western Australia 6150 Australia; 3 Centre for Microscopy, Characterisation and Analysis, The University of Western Australia, 35 Stirling Highway, Perth, Western Australia 6009 Australia

## Abstract

Rhenium and osmium isotopes have been used for decades to date the formation of molybdenite (MoS_2_), a common mineral in ore deposits and the world’s main source of molybdenum and rhenium. Understanding the distribution of parent ^187^Re and radiogenic daughter ^187^Os isotopes in molybdenite is critical in interpreting isotopic measurements because it can compromise the accurate determination and interpretation of mineralization ages. In order to resolve the controls on the distribution of these elements, chemical and isotope mapping of MoS_2_ grains from representative porphyry copper-molybdenum deposits were performed using electron microprobe and nano-scale secondary ion mass spectrometry. Our results show a heterogeneous distribution of ^185,187^Re and ^192^Os isotopes in MoS_2_, and that both ^187^Re and ^187^Os isotopes are not decoupled as previously thought. We conclude that Re and Os are structurally bound or present as nanoparticles in or next to molybdenite grains, recording a complex formation history and hindering the use of microbeam techniques for Re-Os molybdenite dating. Our study opens new avenues to explore the effects of isotope nuggeting in geochronometers.

## Introduction

Ore deposits are the main source of metals for society, and their efficient and sustainable exploration requires a precise understanding of the factors that control their distribution within the upper crust. Application of the Re-Os isotopic system has revolutionized ore deposit research since the 1990’s by addressing two of the most critical issues in the development of genetic models and strategic exploration plans: the source of metals and the age of mineralization^1–5^.

Rhenium 187 is radioactive and decays to radiogenic ^187^Os by beta emission. The Re-Os system follows the law of radioactivity where the total number of ^187^Os atoms in the sample at the present time is equal to the number of atoms of ^187^Os incorporated in the sample at the time of mineral formation and the ^187^Os atoms produced by decay of the ^187^Re parent radionuclide. Due to their chalcophile affinity and behavior during partial melting of the mantle, Re and Os will be concentrated in sulphide phases usually at low ppb and ppt levels, respectively. However, molybdenite (MoS_2_) the most common molybdenum ore mineral constitutes a particular case within sulphide minerals because it contains high Re (in the ppm range) and ^187^Os (at ppb levels), but almost no initial or common ^187^Os, hence all ^187^Os in molybdenite is of radiogenic origin (i.e. produced from decay of ^187^Re)^1,2,5^. These unique characteristics explain why Re-Os molybdenite dating using the whole mineral approach is currently the most widely used single mineral geochronometer in ore deposits, where reliable crystallization ages have been obtained by the direct measurement of ^187^Re and ^187^Os concentrations in the mineral. Although the potential of molybdenite as a single-mineral geochronometer was recognized years ago^6,7^, initial studies were hampered by spurious ages that were interpreted as open system behavior of the isotopic system^8,9^. Furthermore, some researchers have suggested that ^187^Re and ^187^Os isotopes are not spatially linked at the micro-scale in molybdenite precluding the use of microbeam methods for Re-Os dating^10–12^. It has been argued that this isotopic decoupling of Re and Os is caused by radiogenic ^187^Os diffusion which may accumulate in crystal deformation sites^11^. Hence, to obtain accurate and reliable ages, whole molybdenite crystals should be analyzed in order to overcome the inferred decoupling^11,12^.

Here we investigate the distribution of Re and Os in molybdenite, the degree of isotopic and chemical zoning of these elements, the formation of Re-, Os-rich domains and particles in or next to molybdenite, and the processes responsible for intracrystalline/intragrain fractionation. Understanding the controls on Re and Os isotope distribution is critical in interpreting the accuracy of isotopic measurements, and thus explain spurious Re-Os ages obtained by microbeam techniques.

To understand the mineralogical form of incorporation (i.e., nanoparticles vs. solid solution) and the parameters that control the distribution and abundances of Re and Os in molybdenite, we investigated a suite of samples from two porphyry Cu-Mo deposits, El Alacrán (Mexico)^2,13^ and Miranda (Chile)^14^. High-resolution imaging, wavelength-dispersive spectroscopy (WDS) elemental and NanoSIMS isotopic mapping provide the first view of the distribution of the Re and Os elements and their respective isotopes at the micro to nanometer scale. The samples were previously analyzed for Re and Os using N-TIMS^2,14^ and were selected because of their high Re and Os content (Supplementary Table 1), which facilitate their detection by EMPA and NanoSIMS.

## Results

### Elemental distribution in molybdenite

**Figure 1:**
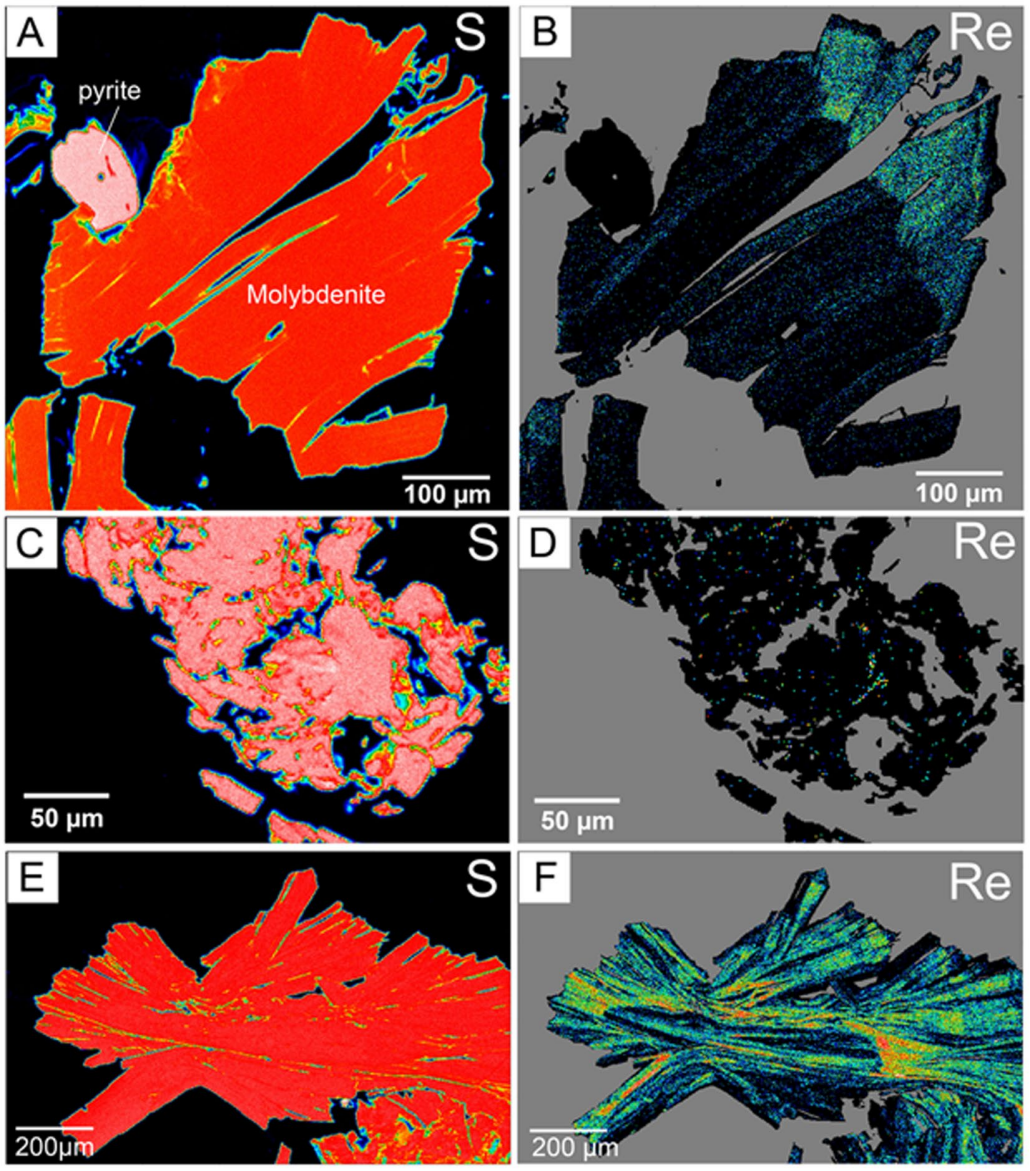
WDS maps for sulfur (right) and rhenium (left) in molybdenite grains. Sulfur distribution is homogeneous in the molybdenite crystal, whereas rhenium shows different patterns of distribution. Warmer colors represent higher concentrations.

Quantitative, wavelength-dispersive (WDS) X-ray compositional maps of Mo, Fe, S, Re, and Os show homogeneous distribution of S and Mo, whereas Re and Os are heterogeneously distributed within molybdenite crystals (Fig. 1 and Supplementary Fig. 1).

Sample Miranda 2569 displays alternating, parallel Re-rich (7,000–9,000 ppm) and Re-poor (1,800–5,000 ppm) zones perpendicular to the growth direction of the *c*-axis (0001) of molybdenite (hexagonal, space group P 6_3_/mmc). The highest (up to 15,000 ppm) relatively homogenous Re concentrations occur as an overgrowth over the primary molybdenite indicating a second Re-rich event of crystallization (Fig. 1B). This overgrowth was formed by a later hydrothermal event and is not evident from routine optical inspection. Rhenium in molybdenite from El Alacrán has a bimodal distribution. In sample Alacrán-B6, Re (700–7,200 ppm) accumulates in discrete micro- to nano-inclusions and or submicron zones (Fig. 1D), whereas in sample Alacrán-B9 rhenium partitions into oscillatory zoning similar to sample Miranda 2569, with primary molybdenite depleted in Re (4,000–8,000 ppm), and secondary molybdenite enriched in the element (10,000–21,500 ppm; Supplementary Data 1). Additionally, high Re concentrations are observed at the edges of the central crystal, indicating overgrowths (Fig. 1F). The pattern is undisturbed by deformation and fragmentation.

The amounts of Os, which were detected in several EMPA analyses in all samples, vary from 400–700 ppm. This particulate distribution combined with single spot maxima on the Os elemental map suggests the presence of submicron Os-bearing inclusions (Supplementary Fig. 1 and Supplementary Data 1).

### Rhenium and osmium isotopes in molybdenite

**Figure 2:**
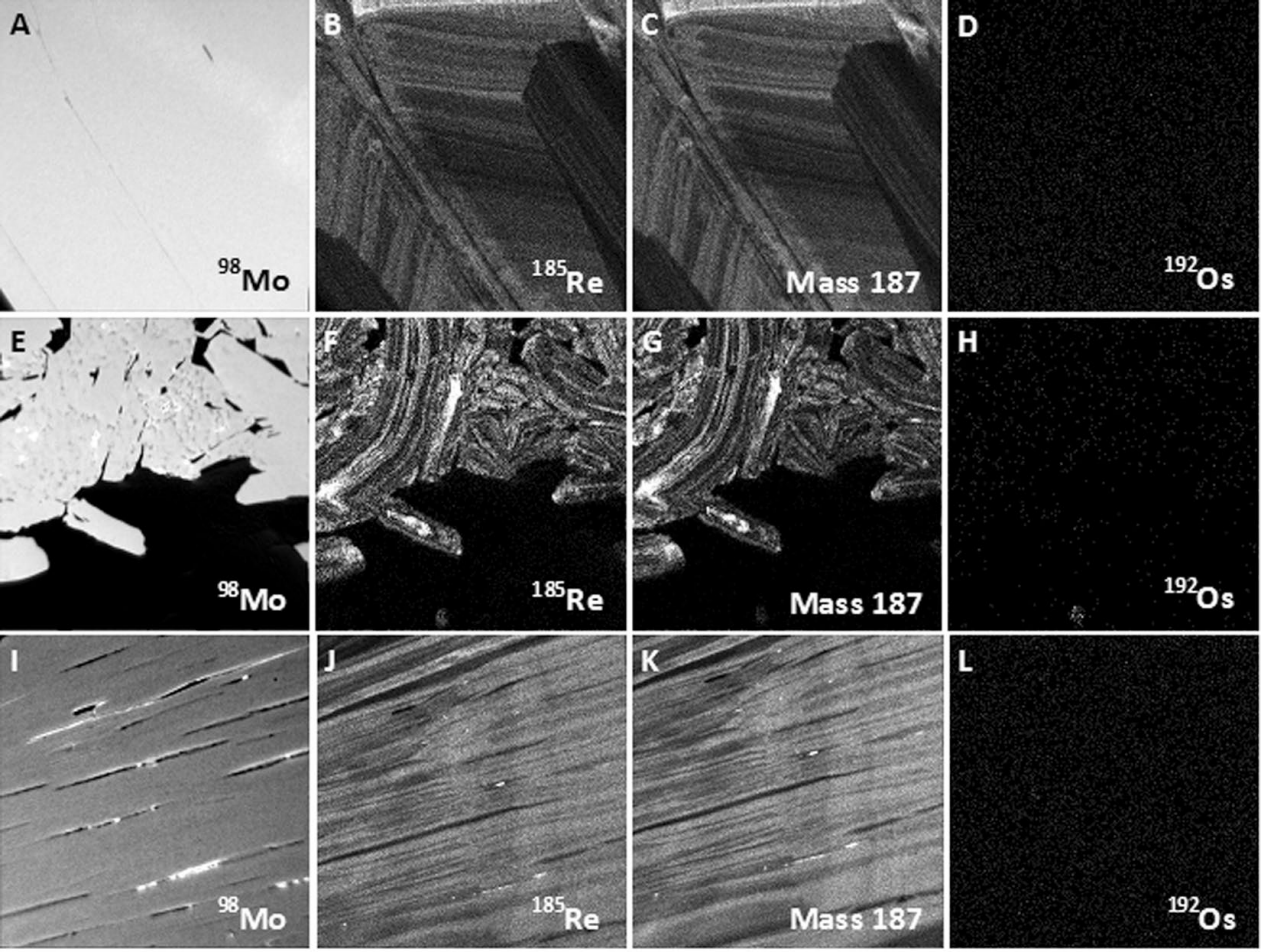
Nano-SIMS isotope maps of selected areas in molybdenite grains. (**A**–**D**) Sample Miranda 2569; (**E–H**) Sample El Alacrán B6; (**I–L**) Sample El Alacrán B9. (**A,E** and **I**) show relative homogeneous distribution of molybdenum mass 98 within molybdenite grains. Distribution of mass ^185^Re (**B,F** and **J**) and mass 187 (^187^Re + ^187^Os) (**C,G** and **K**) show coupled behavior of Re and Os isotopes. (**D,H** and **L**) show homogeneous distribution of common Os (mass 192) in molybdenite grains. Note micro-size Re-Os particle at the bottom of images (**F,G,H**). Images are 50 × 50 µm in size.

**Figure 3:**
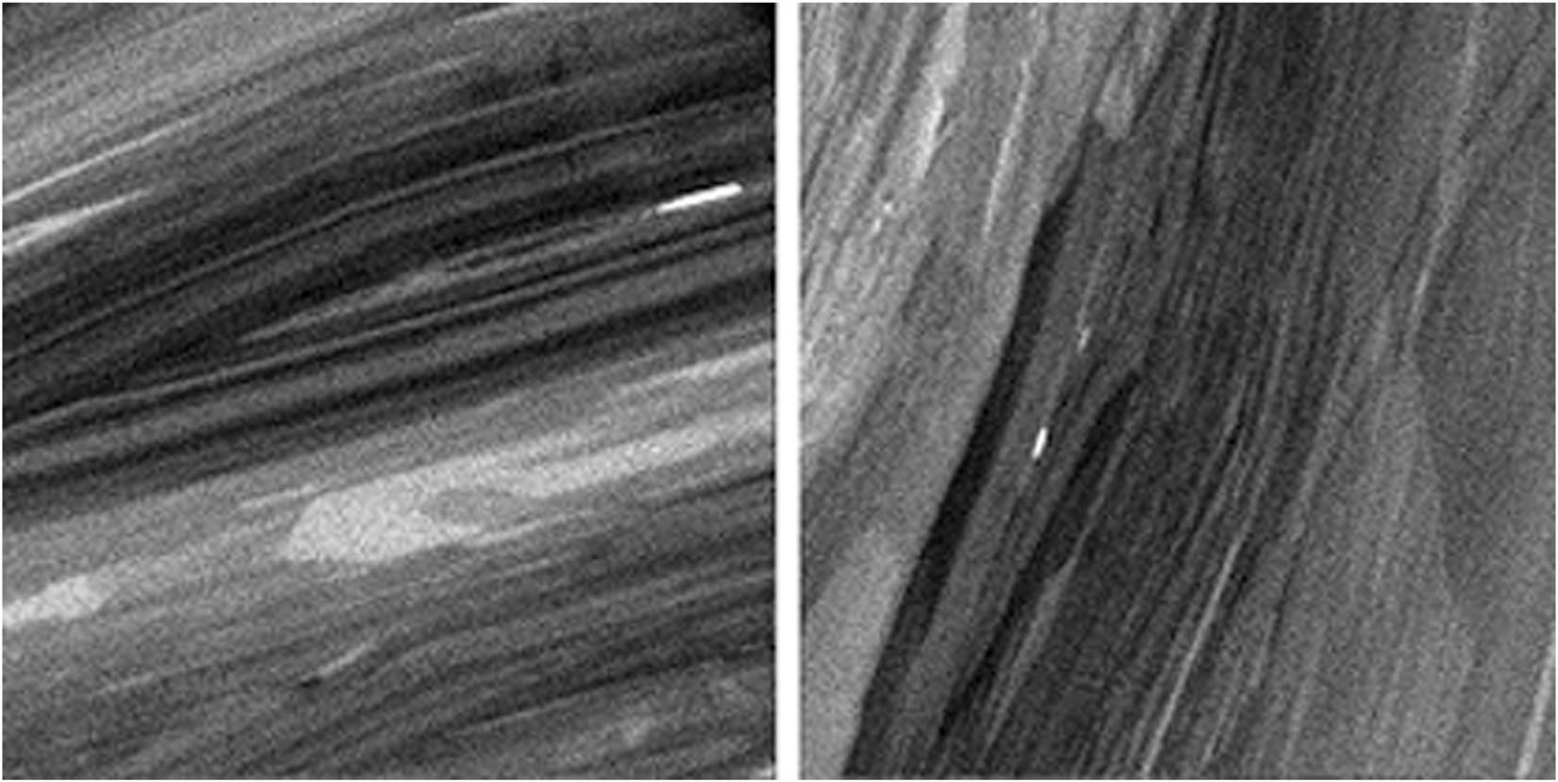
Nano-SIMS ^185^Re isotope maps. The images show oscillatory zoning of Re isotope 185 in sample Alacrán B9. Bright nano-inclusions are possibly rheniite (ReS_2_). Images are 50 × 50 µm in size.

High spatial resolution isotopic mapping of selected areas (50 × 50 μm) included ^98^Mo, ^185^Re, ^192^Os, and mass 187, which represents the combination of the two unresolvable isotopes ^187^Os and ^187^Re (Fig. 2). Iron-(56), ^63^Cu, ^107^Ag isotopes were also monitored in some areas in order to determine mineralogical/isotopic associations with Re and Os. Rhenium-185 isotope map revealed oscillatory zoning in molybdenite, which is present in all analyzed samples, including highly-deformed grains (Fig. 2). All samples show zones with relatively high Re content. Sample Alacrán-B9 hosts Re-rich nano-inclusions (<1 μm in size), which are observed as bright spots in the ^185^Re maps (Fig. 3). These nano-sized inclusions are possibly rheniite (ReS_2_).

**Figure 4:**
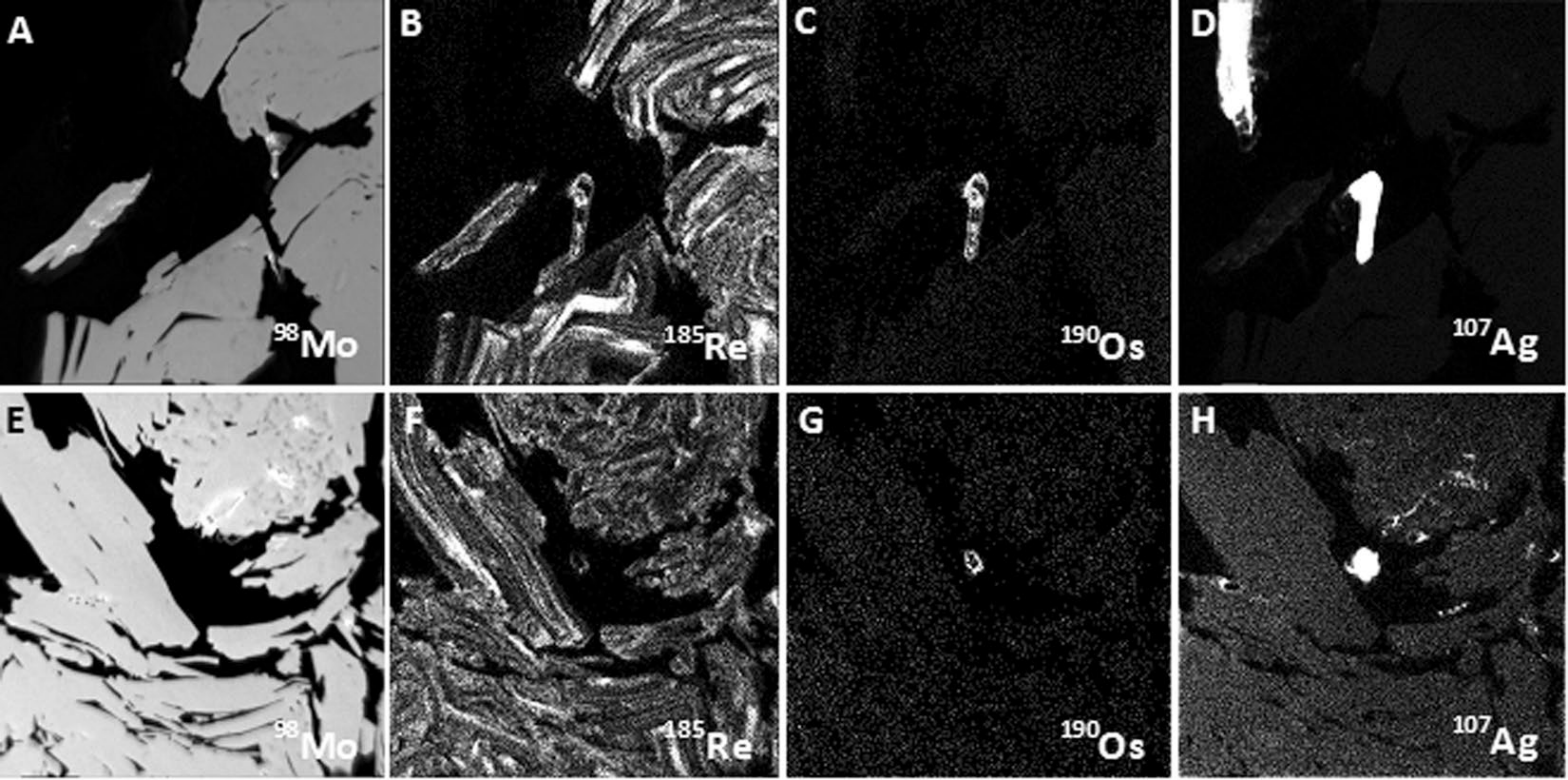
Nano-SIMS isotope maps of selected areas in molybdenite sample Alacrán B6. Images show the homogeneous distribution of ^98^Mo isotope (**A,E**), the oscillatory zoning of ^185^Re (**B,F**), and the presence of Ag-rich Re-Os bearing nano- to micron-size particles attached to (**B**–**D**) or within interstitial space of molybdenite (**F–H**). Images are 50 × 50 µm in size.

Mass 187 maps, which show the distribution of both ^187^Re and ^187^Os isotopes, display an identical oscillatory pattern as ^185^Re (Fig. 2). This indicates that there is no isotopic decoupling between ^187^Re and ^187^Os, as previously suggested^11,12^. Further, ^192^Os distribution maps show that common Os is present in very low abundance in the studied material. Samples Alacrán-B6, -B9, and Miranda 2569 have a uniform ^192^Os isotopic distribution (i.e., structurally bound, Figs. 2D,H,L). Additionally, common Os-bearing nano- to micron-size particles, <1 μm and up to 10 μm in size, are observed attached to or within interstitial space of molybdenite (Figs. 2F–H), some of which are Ag-rich Re-Os nanoparticles (Fig. 4).

## Discussion

### Distribution of noble metals in ore minerals

Heterogeneous Re distribution, including oscillatory zoning, and Re-rich overgrowths in molybdenite crystals have been previously reported^15–17^, although the mechanisms controlling the Re distribution remain poorly understood. Oscillatory mineral zonation has been attributed to intrinsic, extrinsic or epigenetic processes^18–20^, whereas overgrowths are clearly associated with changes in the Re budget of the mineralizing fluid. The presence of Re-Os nanoparticles in molybdenite questions the incorporation of noble metals in sulphide minerals.

The controls of incorporation and concentration of noble metals (Os, Ir, Ru, Rh, Pt, Pd, Au, Ag and Re) in hydrothermal ore minerals remain uncertain mainly because they are usually present at very low concentrations (low ppt levels) and occur at the micro to nano-scale. Such properties impose even greater difficulties when investigating isotope geochemistry. The observed partitioning of Re and Os into solid-solution, and/or to nano-to-micro size zones and domains, and formation of metal particles is the first empirical evidence for the bimodal behavior of Os and Re within molybdenite.

Most studies on the distribution of noble metals have focused on magmatic ore deposits where discrete micron-size inclusions of platinum-group elements (PGEs) have been observed in chromite grains from ophiolites, layered mafic intrusions and in base metal sulphides from Cu-Ni magmatic deposits^21,22^. These works showed that PGEs are present in solid solution and as microparticles within sulphides^22^. Similarly, studies on the distribution of gold and silver in hydrothermal sulphides show that these elements can be present in solid solution and as micro- to nano-size particles^23–25^. Our observations show that both Re and Os also occur in solid solution and as discrete nanoparticles, providing indisputable evidence for the concentration of Os and Re as nano-clusters or “nuggets” within the host mineral.

The incorporation of noble metals and perhaps some other trace elements within mineral phases appears to be mostly heterogeneous, in several cases forming nanoparticles or nanoclusters, which can affect the accurate measurement of elemental concentrations or isotopic signatures by microbeam techniques.

### Controls on the incorporation of Re and Os in molybdenite

Rhenium and Os are heterogeneously distributed reflecting changes in the composition of the hydrothermal fluid. Direct observation of (i) oscillatory isotopic and chemical nano-zoning of rhenium, (ii) Re-rich overgrowths, and (iii) presence of common Os in domains in molybdenite grains and in the associated nanoparticles provide an explanation for spurious Re-Os ages obtained by laser ablation ICP-MS. Alternating incorporation of Re into molybdenite during growth (Fig. 1) is most probably caused by variations in the Re budget of the hydrothermal fluid, produced by changes in temperature, pH, ligand concentration and oxidation state of the hydrothermal fluid^26,27^ that can occur over short periods at a scale of tens to hundreds or even thousands of years. These factors can also control the formation of Os-Re nanoparticles by Ostwald ripening, as seen for example for Au in arsenian pyrite and Os-Ir alloys in laurite^23,24^. Overgrowths are formed by a later hydrothermal event and in some cases are not evident under petrographic inspection. Overgrowths in molybdenite crystals might be a common phenomenon in ore deposits caused by superimposed hydrothermal events. Whole mineral age determination may overcome this distribution-related limitation, if in fact the mineral has formed within a restricted time frame of less than a few hundred thousand years, and no disturbance from isotopically different areas are present in the studied material. Otherwise, the ages obtained by this whole mineral approach represent an average.

By revealing common Os in solid solution and in particles in molybdenite we prove that common Os is incorporated into the molybdenite structure and/or is tightly bounded to the mineral surface. However, it accumulates in relatively low concentration in comparison with radiogenic Os. Additionally, the ^187^Os component of common Os is ca. 1.5% of the total amount of common Os present, hence it can be considered negligible for the age calculation.

### Parent-daughter isotope decoupling

Published studies have shown diffusion of radiogenic Pb in the zircon structure and formation of Pb nanoparticles^28–30^. This decoupling of radiogenic Pb from parent U has a profound effect on the interpretation of U-Pb ages obtained by microbeam analyses. A similar diffusion-driven decoupling of Os and Re was proposed for radiogenic ^187^Os in molybdenite^9–11^. However, the observed/detected Os nanoparticles (Figs. 2 and 4) in the studied samples contain common Os, not radiogenic ^187^Os.

NanoSIMS mapping indicates that ^187^Re and radiogenic ^187^Os are not decoupled in molybdenite (Fig. 2). In fact, no accumulation of ^187^Os was identified in the samples. Thus, there is no evidence of post-crystallization diffusion of ^187^Os in the molybdenite structure, as it has been documented for radiogenic Pb in zircon^28,29^. Furthermore, the distribution of ^185^Re and mass 187 (i.e., ^187^Re + ^187^Os) indicates that there is no fractionation of Re isotopes within molybdenite. However, our observations reveal that Re is heterogeneously distributed within molybdenite, and common Os present as solid solution does not correlate with the distribution of Re isotopes (Fig. 2). The heterogeneous distribution of Re and Os in molybdenite at the nano-scale precludes the use of microbeam techniques for dating purposes.

Therefore, understanding the mechanisms than control the distribution and abundances of parent and daughter isotopes and their form of incorporation in any given mineral, including their potential occurrence as nanoparticles or metal clusters, is of utmost importance in the interpretation of isotopic measurements and of geochronological ages.

Our study contributes to the growing evidence that heterogeneous distribution of trace elements may be a more common phenomenon in minerals than previously assumed. Understanding of the mechanisms that control isotopic coupling or decoupling within single mineral grain/crystal is of critical importance for the unbiased interpretation of geochemical data and isotopic ages.

## Methods

### Electron probe microanalyses (EPMA)

Chemical composition of molybdenite was analyzed using JEOL JXA8530F electron microprobe equipped with 5 wavelength dispersive spectrometers. The operating conditions were 40 degrees take-off angle, beam energy of 20 keV, beam current of 20 nA, and the beam diameter of 1 µm. Elements were acquired using analyzing crystals PETJ for S Kα and Mo Lα, PETH for Pb Mα, Bi Mα, LiF for Fe Kα, Cu Kα, Te Lα, and W Lα, LiFH for V Kα, Re Lα, Os Lα, and TAP for Se Lα. The standards used for instrument calibration were molybdenite for Mo and PETJ S Kα, Bi_2_Se_3_ for Se Lα, Bi metal for Bi Mα, magnetite for Fe Kα, Cu metal for Cu Kα, scheelite for W Lα, Re metal for Re Lα, Os metal for Os Lα, and Pb metal for Pb Mα. The on-peak counting time was 20 seconds for all elements and Mean Atomic Number (MAN) background corrections used throughout^31^. The sample and standard intensities were corrected for deadtime. Unknown and standard intensities were corrected for dead time and the ZAF algorithm was used for matrix absorption^32^ and data reduction used the Probe for EPMA software package. On-peak interference corrections were applied as appropriate^33^. Detection limits ranged from 0.008 weight percent for V Kα to 0.038 weight percent for W Lα.

Quantitative wavelength-dispersive spectrometry (WDS) X-ray maps were collected utilizing aforementioned calibration set up. Detection limit maps were acquired for these elements and applied as the minimum cut-off values. Map acquisition utilized a 100-nA beam current with variable pixel dimension and variable m/s dwell time per pixel. Data were processed using the Calcimage software package and output to Surfer® for further processing and enhancement.

### NanoSIMS analyses

Elemental and isotopic mapping was carried out using a CAMECA NanoSIMS 50 L at the University of Western Australia. The analysis was performed with an O^−^ beam generated by a Hyperion (H200) RF plasma oxygen ion source. The beam current was approximately 17 pA and the spot size was approximately 100 nm. Each area of interest was pre-sputtered with the primary beam to a dose of >1 × 10^17^ ions/cm^2^. Due to the geometry of the mass spectrometer, it was not possible to collect all the relevant isotopes simultaneously, thus each area was mapped twice using two different configurations of the multicollection system. The magnetic field was fixed, and the electron multiplier (EM) detectors were positioned to collect signal from ^56^Fe, ^63^Cu, ^98^Mo, ^107^Ag, ^185^Re, ^190^Os during the first run, and then the last two detectors were moved to collect ^187^Re and ^192^Os during the second run. The peak positions were calibrated using pure Re and Os metal standards. As sensitivity was a key issue and there were no significant mass interferences, no slits were used in the mass spectrometer.

Images were acquired with a raster size of 45 or 50 μm^2^, at a resolution of 512 × 512 pixels, with a dwell times of 25 or 30 ms/pixel. Maps were corrected for 44 ns deadtime on each individual pixel. Images were processed using the OpenMIMS plugin for FIJI/ImageJ (https://github.com/BWHCNI/OpenMIMS).

## Electronic supplementary material

Supplementary Information

Supplementary Information Dataset 1
