# Supplementary material for: Dissecting the Re-Os molybdenite geochronometer
Source: Sci Rep. 2017 Nov 22;7:16054. doi: 10.1038/s41598-017-16380-8 (PMC5700062; doi:10.1038/s41598-017-16380-8)
Supplement: Supplementary file 1 — Supplementary Information [file 41598_2017_16380_MOESM1_ESM.pdf]

Supplementary Information for

Dissecting the Re-Os molybdenite geochronometer

Fernando Barra[1*], Artur Deditius[2], Martin Reich[1], Matt R. Kilburn[3], Paul Guagliardo[3], Malcolm P. Roberts[3]

correspondence to: fbarrapantoja@ing.uchile.cl

This PDF file includes:

Supplementary Figure 1
Supplementary Table 1
Caption for Supplementary Data 1

Other Supplementary Information for this manuscript includes the following:

Supplementary Data 1 (Excel file)

Sample Miranda 2569

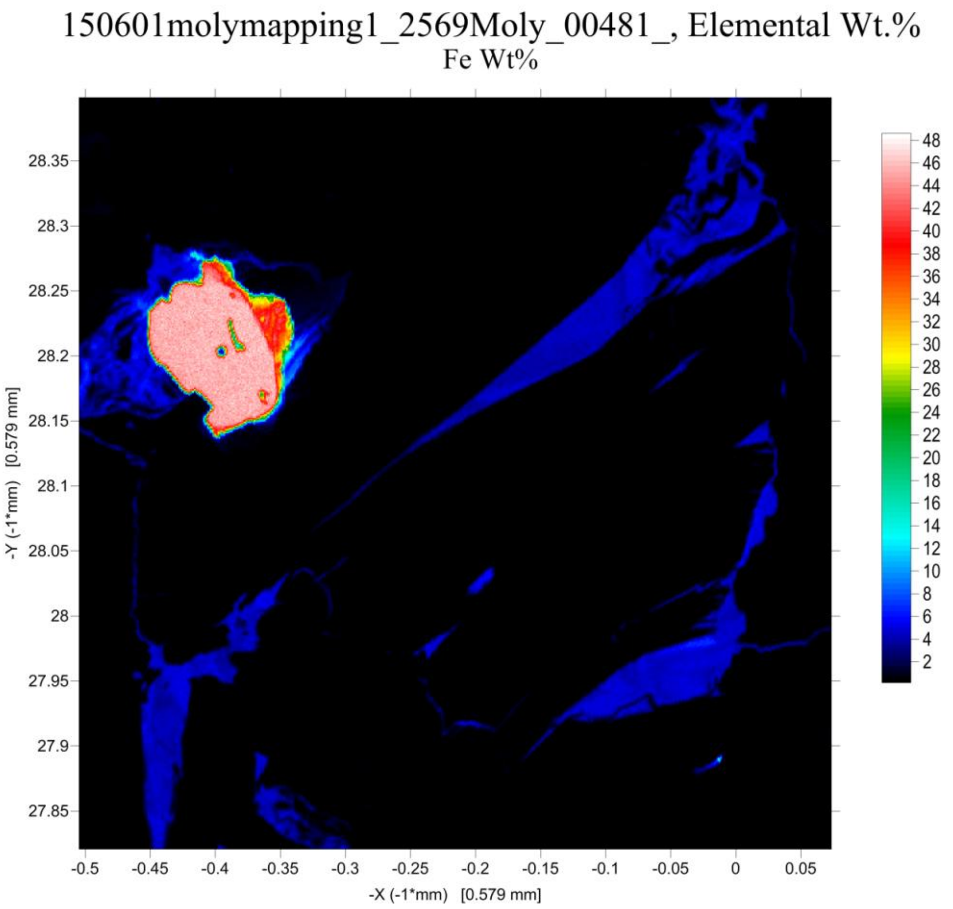

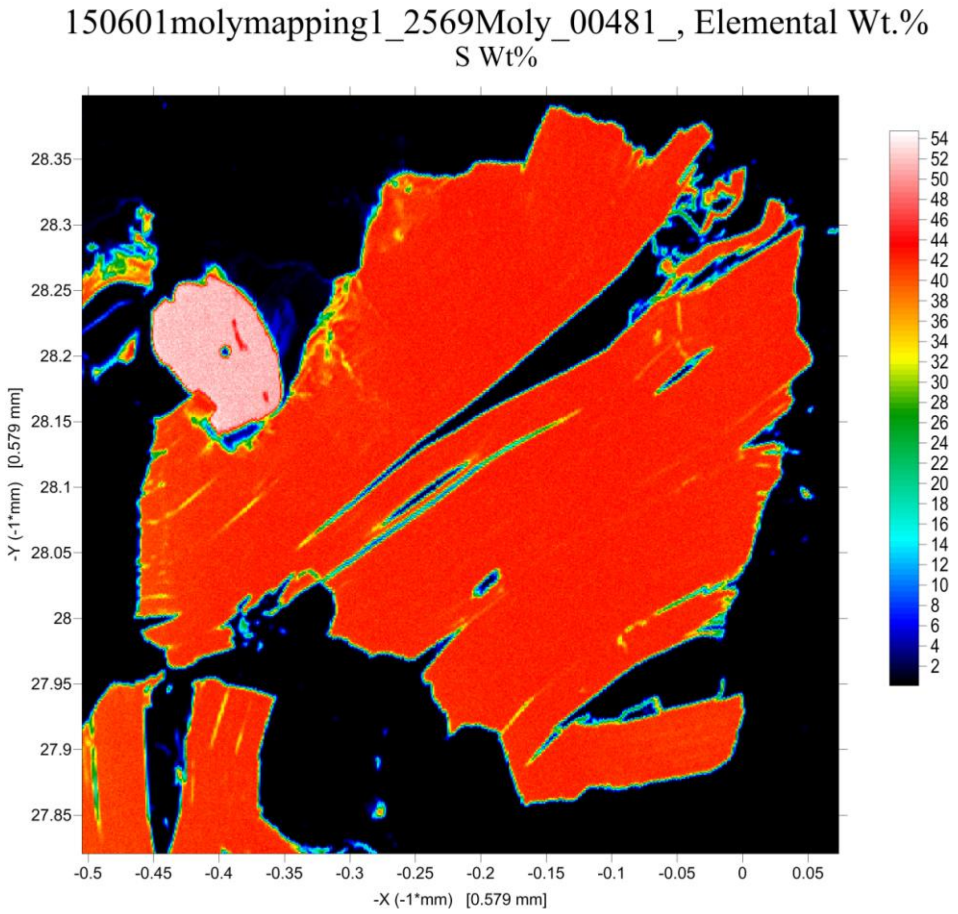

150601molymapping1_2569Moly_00481_, Elemental Wt.%
Re Wt%

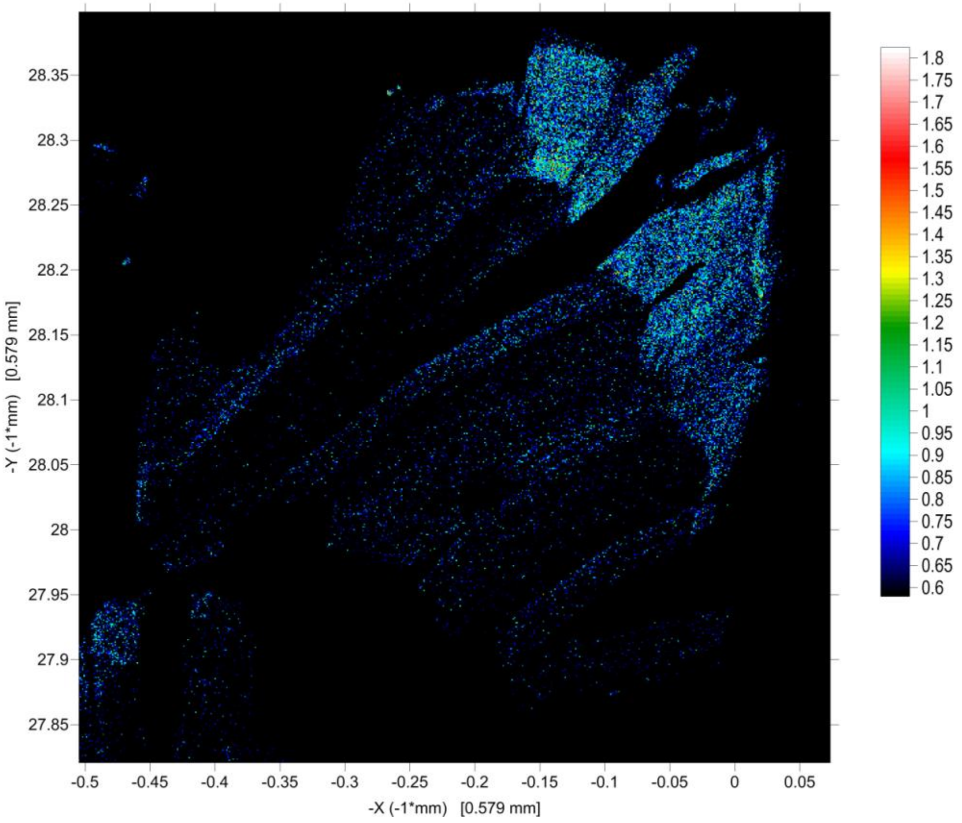

150601molymapping1_2569Moly_00481_, Elemental Wt.%
Os Wt%

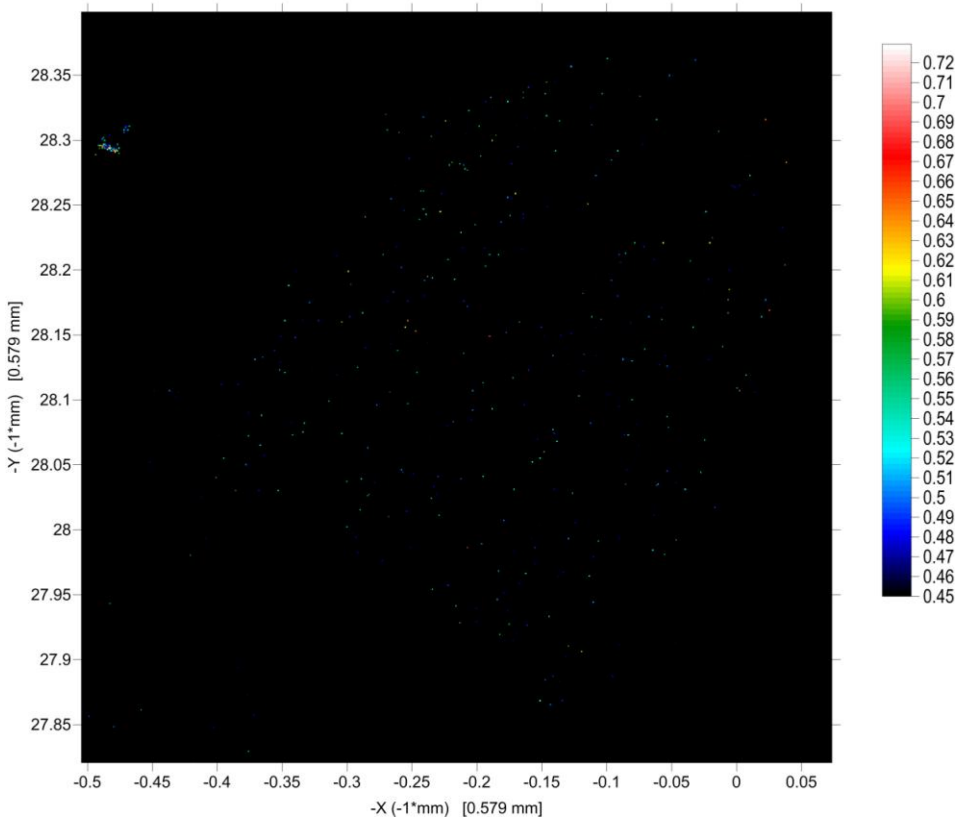

Sample Alacrán-B6

150601molymapping1_B6Molya_00483_, Elemental Wt.%

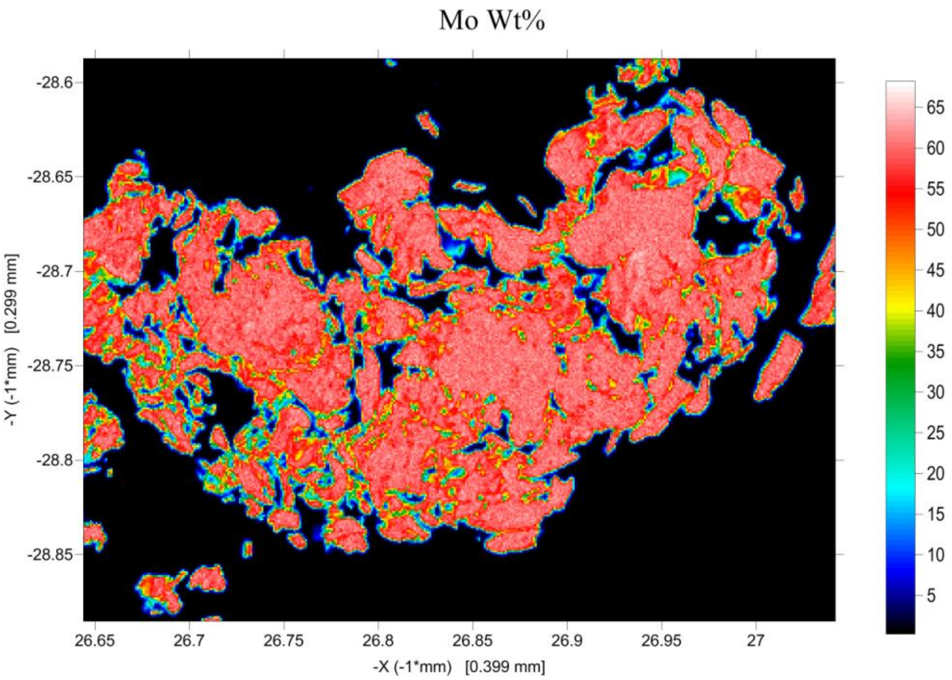

150601molymapping1_B6Molya_00483_, Elemental Wt.%

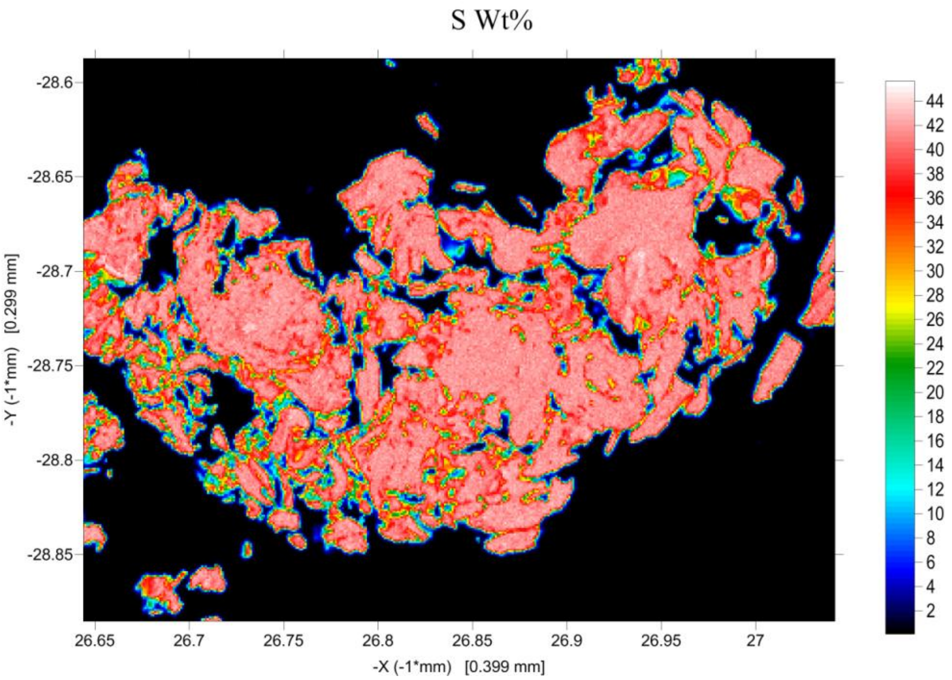

150601molymapping1_B6Molya_00483_, Elemental Wt.%

Re Wt%

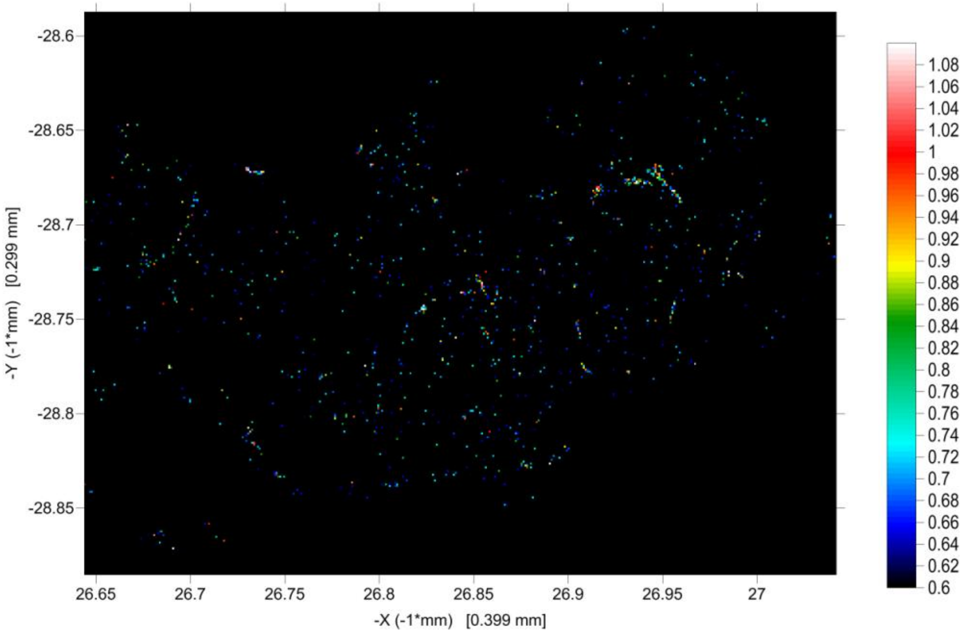

150601molymapping1_B6Molya_00483_, Elemental Wt.%

Os Wt%

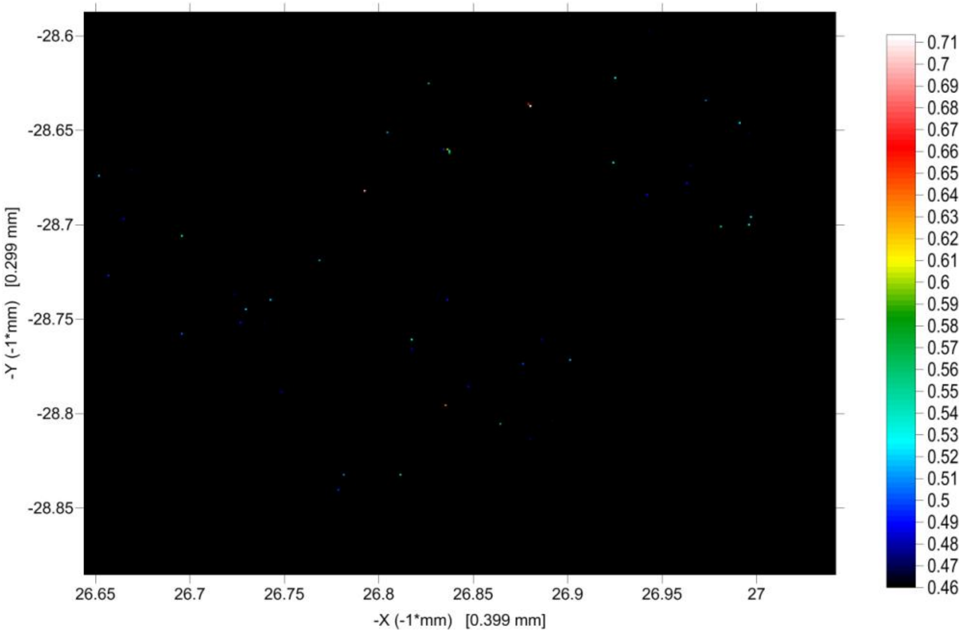

Sample Alacrán-B9

150601molymapping1_B9Moly_00482_, Elemental Wt.%
Fe Wt%

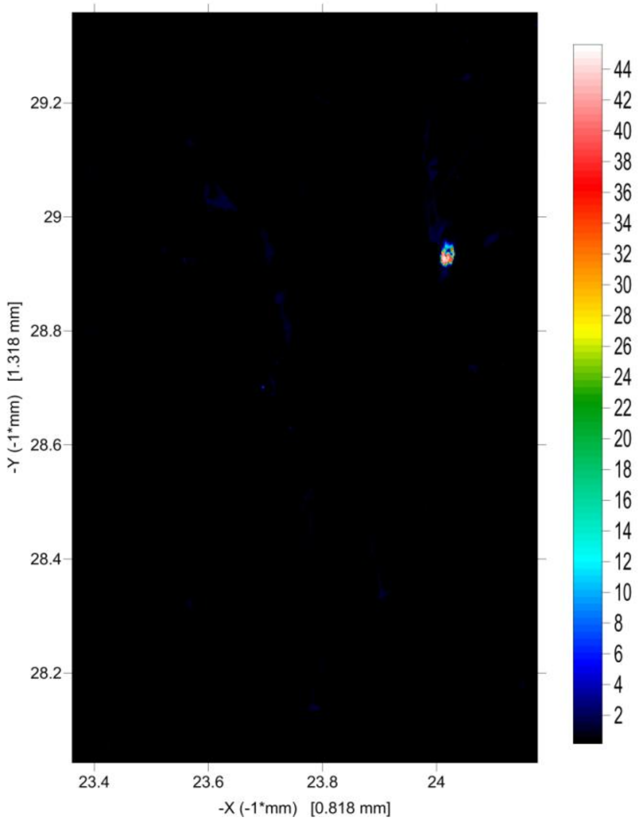

150601molymapping1_B9Moly_00482_, Elemental Wt.%
S Wt%

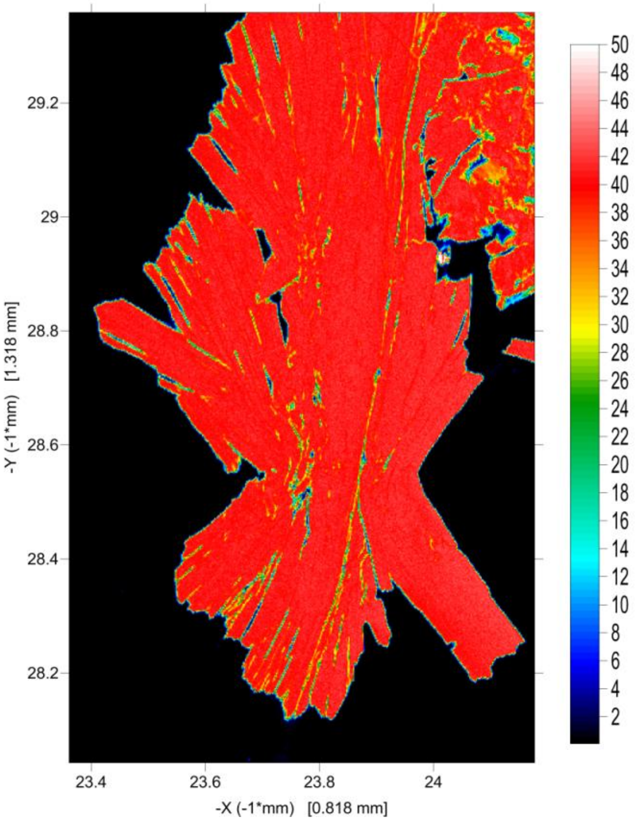

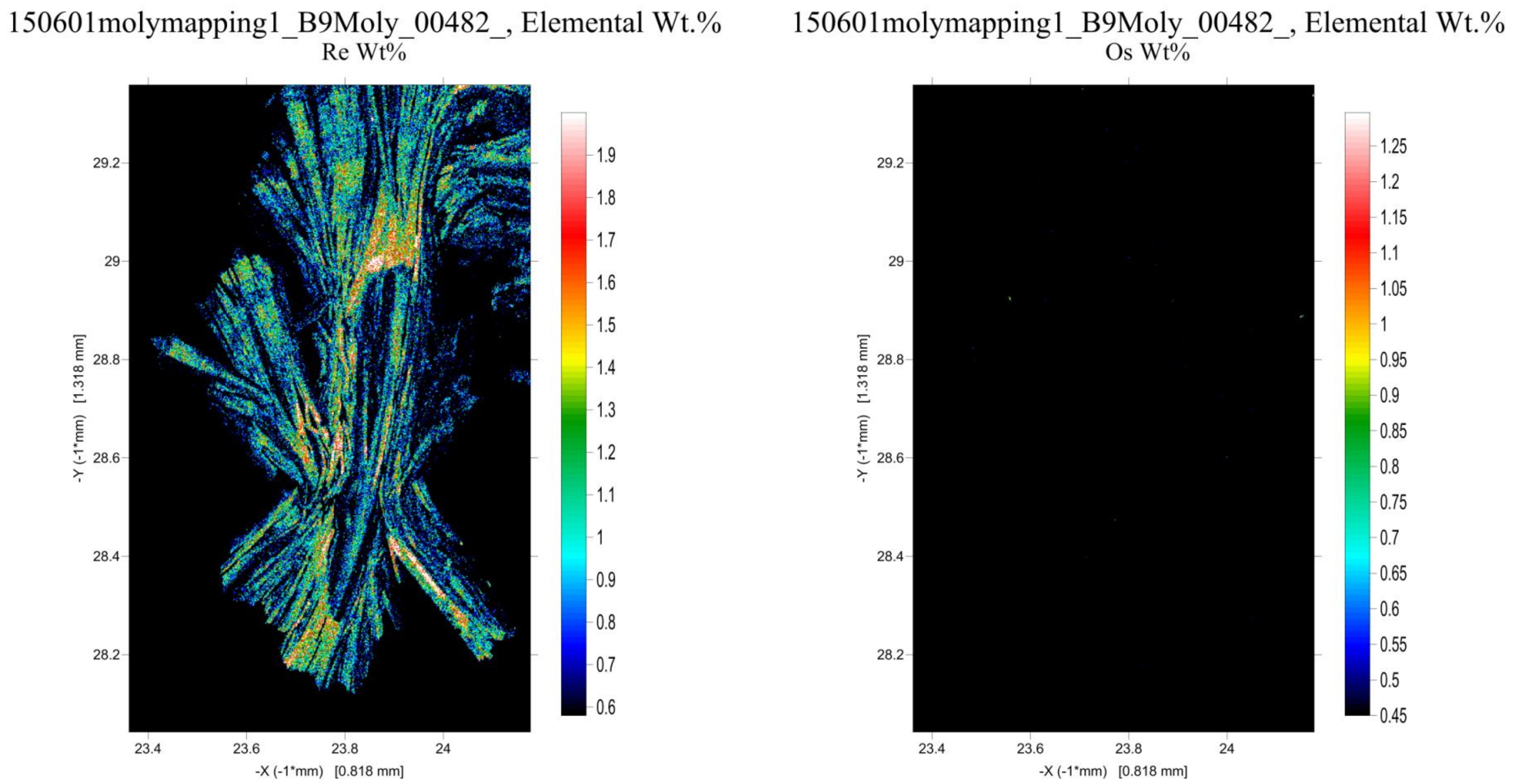

Figure 1. WDS maps for sulfur, iron, molybdenum, rhenium and osmium in molybdenite grains. Sulfur distribution is homogeneous in the molybdenite crystals, whereas rhenium shows different patterns of distribution. Osmium is only observed as small scarce microinclusions.

Supplementary Table 1.

Reported Re-Os data for molybdenite samples studied by EMPA and NanoSIMS.

Deposit	Sample Name	Total Re (ppm)	^{187}Re (ppm)	Total Os (ppb)	Age (Ma)	Reference
MIRANDA, CHILE	2569	2858.4	1789.3	1087.6	36.5 ± 0.2	*(14)*
EL ALACRAN, MEXICO	B9	7352	4622	4690	60.9 ± 0.2	*(2,13)*
EL ALACRAN, MEXICO	B6	10424	6553	6641	60.8 ± 0.2	*(2,13)*

Supplementary Data 1 (separate Excel file)

Concentrations of trace elements measured by EMPA for the studied molydenite samples.
